# Regulation of DNA replication and chromosomal polyploidy by the MLL-WDR5-RBBP5 methyltransferases

**DOI:** 10.1242/bio.019729

**Published:** 2016-10-15

**Authors:** Fei Lu, Xiaojun Wu, Feng Yin, Christina Chia-Fang Lee, Min Yu, Ivailo S. Mihaylov, Jiekai Yu, Hong Sun, Hui Zhang

**Affiliations:** 1 Laboratory of Chemical Genomics, School of Chemical Biology and Biotechnology, Peking University Shenzhen Graduate School, Shenzhen, Guangdong 518055, China; 2 Basic Science Division, Nevada Cancer Institute, Las Vegas, NV 89135, USA; 3 Department of Chemistry and Biochemistry, University of Nevada, Las Vegas, NV 89154, USA

**Keywords:** DNA replication, H3K4 methylation, WDR5, RBBP5, Re-replication

## Abstract

DNA replication licensing occurs on chromatin, but how the chromatin template is regulated for replication remains mostly unclear. Here, we have analyzed the requirement of histone methyltransferases for a specific type of replication: the DNA re-replication induced by the downregulation of either Geminin, an inhibitor of replication licensing protein CDT1, or the CRL4CDT2 ubiquitin E3 ligase. We found that siRNA-mediated reduction of essential components of the MLL-WDR5-RBBP5 methyltransferase complexes including WDR5 or RBBP5, which transfer methyl groups to histone H3 at K4 (H3K4), suppressed DNA re-replication and chromosomal polyploidy. Reduction of WDR5/RBBP5 also prevented the activation of H2AX checkpoint caused by re-replication, but not by ultraviolet or X-ray irradiation; and the components of MLL complexes co-localized with the origin recognition complex (ORC) and MCM2-7 replicative helicase complexes at replication origins to control the levels of methylated H3K4. Downregulation of WDR5 or RBBP5 reduced the methylated H3K4 and suppressed the recruitment of MCM2-7 complexes onto replication origins. Our studies indicate that the MLL complexes and H3K4 methylation are required for DNA replication but not for DNA damage repair.

## Abstract (precis)

**Summary:** DNA replication or re-replication of DNA induced after loss of Geminin or CLR4^CDT2^ is regulated by the methylation activities of the MLL-WDR5-RBBP5 methyltransferases on histone H3 at lysine 4 (H3K4).

## INTRODUCTION

In eukaryotic cells, the chromosomal DNA replicates once in a single cell cycle in a temporally regulated manner (Arias and Walter, 2007; Blow and Dutta, 2005). How the chromatin template structure is regulated for the initiation of DNA replication at replication origins remains unclear (Arias and Walter, 2007; Chadha and Blow, 2010; Kuo et al., 2012). In the cell cycle, the initial step for DNA replication involves the formation of the pre-replicative complex (pre-RC) on DNA replication origins in late mitosis and early G1 phase (Arias and Walter, 2007). The pre-RC formation involves the sequential assembly of the origin recognition complex (ORC), composed of six ORC proteins (ORC1-6), CDC6, and CDT1 onto specific DNA replication origins (Blow and Dutta, 2005). The licensing process of the replication origins further requires the recruitment of the minichromosome maintenance protein complex (MCM), consisting of six MCM proteins (MCM2-7) that form a replicative helicase complex, onto chromatin for the next round of DNA replication (Remus et al., 2009). In metazoans, a critical regulation that prevents DNA re-replication at replication origins in a cell cycle is mediated through Geminin, a negative regulatory protein that directly binds to CDT1 to inhibit the key licensing activity of CDT1 for replication initiation (Blow and Dutta, 2005; McGarry and Kirschner, 1998; Melixetian and Helin, 2004; Mihaylov et al., 2002; Wohlschlegel et al., 2000; Zhu et al., 2004). Downregulation of Geminin is sufficient to activate CDT1 and consequently promotes the initiation of DNA re-replication, producing a cell with an enlarged polyploid nucleus with more than 4N DNA content (Melixetian et al., 2004; Mihaylov et al., 2002; Zhu et al., 2004). Another critical mechanism that prevents the re-replication of replicated origins is mediated through the degradation of CDT1 by an ubiquitin E3 ligase complex, CLR4^CDT2^, composed of CUL4, RBX1 (ROC1), DDB1, and a WD40 protein CDT2 (also called L2DTL or DTL) (Higa et al., 2006a,b; Jin et al., 2006), once DNA replication initiates in S-phase or in response to DNA damage. Depletion of CDT2 stabilizes the CDT1 protein in S-phase and consequently induces re-licensing of replication origins, re-replication, and formation of a partially polyploid nucleus (Higa et al., 2006a; Jin et al., 2006).

Increasing lines of evidence suggest that the initiation of DNA replication is regulated by chromatin structure (Groth et al., 2010; Havens and Walter, 2011; Miotto and Struhl, 2010). Recent reports show that DNA replication origins are located at specific chromatin regions with unique histone modifications (Kuo et al., 2012; Tardat et al., 2010). A SET-domain containing histone methyltransferase, SET8 (Pre-SET7), mono-methylates lysine 20 of histone H4 (H4K20), and this histone modification mediates the specific interaction between ORC1 and H4K20-methylated replication origins (Kuo et al., 2012; Tardat et al., 2010; Vermeulen et al., 2010). Replication licensing is also regulated by HBO1, a MYST histone acetylase that binds to CDT1 and acetylates histone H4 at K5, K8, and K12 (Miotto and Struhl, 2008, 2010). These acetylated lysines and HBO1 have been located at several replication origins, including the one at the human *Mcm4* gene (Miotto and Struhl, 2008, 2010).

Previous studies have shown that the actively transcribed chromatin regions appear to replicate DNA early in S-phase (Karnani et al., 2010; Rampakakis et al., 2009). In eukaryotes, the transcriptionally active chromatin regions are usually enriched with trimethylated lysine 4 (K4) in histone H3 (H3K4) (Greer and Shi, 2012; Martin and Zhang, 2005). The MLL histone methyltransferase complexes, each composed of a member of the MLL SET-domain protein family, and other essential components including ASH2L, DPY30, and WD40 proteins WDR5 and RBBP5, catalyze the mono- and tri-methylations of H3K4 (Greer and Shi, 2012; Higa et al., 2006b; Jiang et al., 2011; Martin and Zhang, 2005; Wysocka et al., 2005). In this report, we show that the MLL-WDR5-RBBP5 methyltransferase complexes and H3K4 methylation are required for DNA replication in human cells.

## RESULTS

### Reduction of WDR5 suppresses DNA re-replication in Geminin-deficient cells

**Fig. 1.**
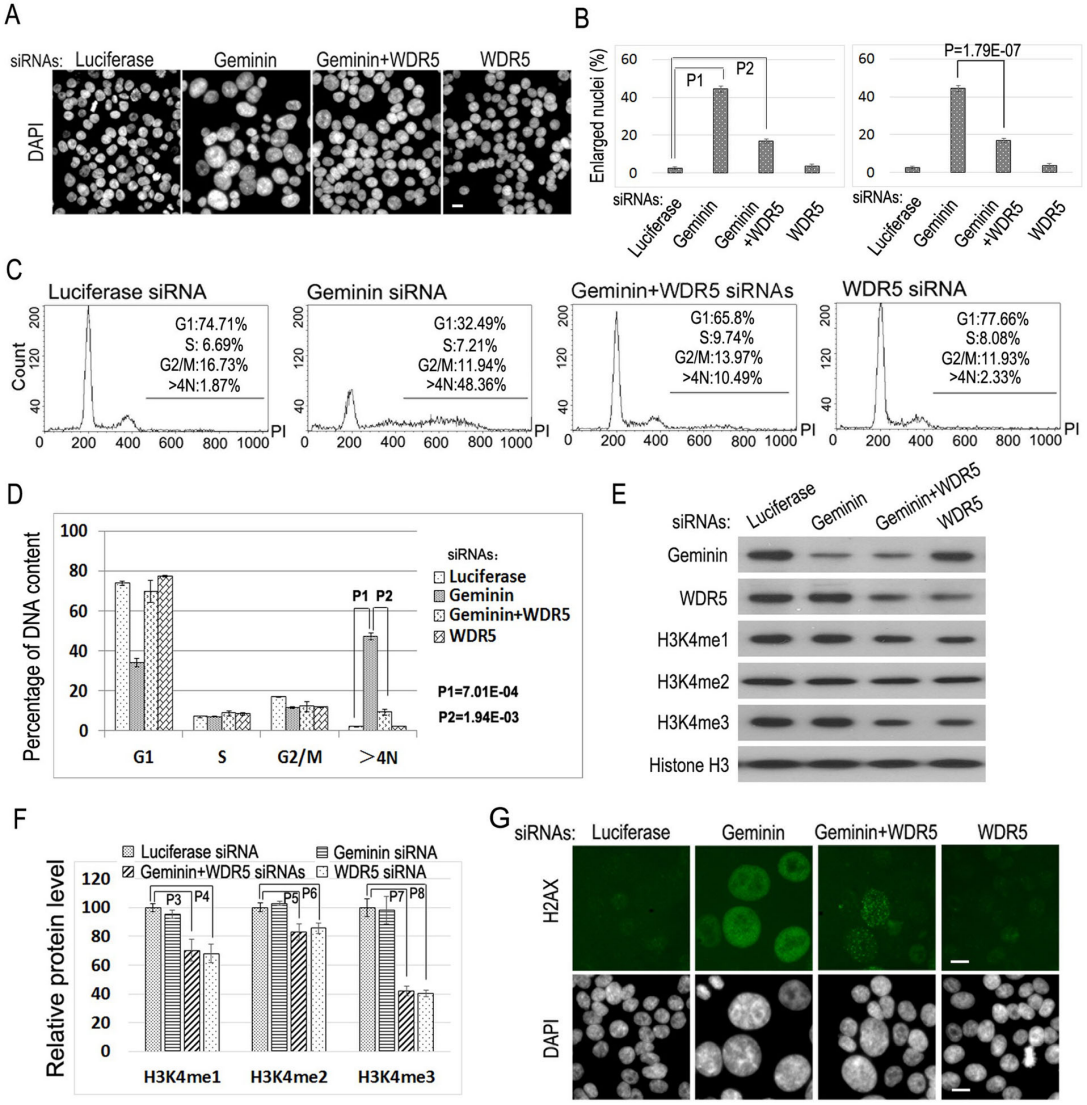
**Reduced expression of WDR5 suppresses re-replication induced by Geminin deficiency.** (A) HCT116 cells were transfected with 50 nM siRNAs of luciferase (control), Geminin, Geminin+WDR5, and WDR5 for 48 h. The cells were fixed with paraformaldehyde and nuclei were stained with DAPI. Scale bar: 50 μm. (B) The percentages of enlarged nuclei in A. The percentages of enlarged nuclei relative to normal nuclei in each sample in A were quantified in five different image fields as described in the Materials and Methods with error bars indicating the standard deviation. Left plot: the statistical differences in enlarged nuclei between control siRNA (Luciferase)-treated and each specific siRNA-treated cells were calculated using the two-tailed Student's *t*-test (*P*1=4.34E-09, *P*2=3.53E-07). Right plot: significant difference was observed between Geminin siRNA-treated and Geminin+WDR5 double siRNA-treated cells which was evaluated by the two-tailed Student's *t*-test (*P*=1.79<0.01). (C) FACS analysis of DNA contents in A. The polyploid cell populations (>4N) are indicated. Three independent experimental repeats were conducted with similar results, and only one representative experiment is shown. (D) The statistical differences in polyploid DNA (>4N) in Geminin siRNA-treated and Geminin+WDR5 double siRNA-treated cells were calculated using the two-tailed Student's *t*-test (*P*1=7.01E-04, *P*2=1.94E-03). (E) The proteins in the lysates of cells in A were analyzed by immunoblotting with specific antibodies as indicated to monitor changes in H3K4 methylation. (F) The alterations of histone H3K4 methylations in D were quantified. The error bars indicate standard deviation of triplicated samples. The statistical differences of histone H3K4 methylations between control and specific siRNA samples were analyzed using the two-tailed Student's *t*-test (*P*3=0.00956, *P*4=0.00575, *P*5=0.0279, *P*6=0.0171, *P*7=8.42E-04 and *P*8=6.56E-04). (G) Immunofluorescence staining of H2AX in A. The cells were incubated with the anti-phospho-histone H2AX (Ser139) antibody and the FITC-conjugated secondary antibody (green). Nuclear DNA was counter-stained with DAPI. Scale bars: 50 μm.

Since DNA re-replication in a single eukaryotic cell cycle would lead to chromosome polyploidy and genome instability (Blow and Dutta, 2005), we investigated the potential involvement of histone modification in DNA replication by analyzing this specific type of DNA replication. In metazoans, both Geminin and CRL4^CDT2^ negatively and independently regulate the replication licensing activity of CDT1 for DNA replication (Higa et al., 2006b; Jin et al., 2006; Mihaylov et al., 2002). We examined whether DNA re-replication induced by abnormal activation of CDT1 is regulated by histone modification in human colorectal cancer HCT116 cells that contain a pseudo-diploid genome (Ballabeni et al., 2004). Reduced expression of Geminin by specific siRNAs activates CDT1 and consequently induces chromosomal DNA re-replication (Ballabeni et al., 2004; Mihaylov et al., 2002; Zhu et al., 2004), promoting the formation of enlarged nuclei that contain more than 4N DNA content (Fig. 1A-D). Downregulation of Geminin also caused prominent nuclear staining of H2AX (Fig. 1G), indicating the activation of the replication/DNA damage checkpoints by the presence of elongation forks during DNA re-replication (Ballabeni et al., 2004; Jin et al., 2006). We found that siRNA-mediated reduction of WDR5, a key component of the MLL-WDR5-RBBP5 methyltransferase complexes that mono- and trimethylate H3K4 (Wysocka et al., 2005), led to the marked reduction on the formation of enlarged nuclei caused by Geminin deficiency (Fig. 1A-F). Reduction of WDR5 also dramatically decreased the number of cells that were positive for H2AX staining in Geminin-deficient cells (Fig. 1G). Flow cytometry (FACS) analyses also revealed that depletion of WDR5 eliminated the percentage of cells that contain >4N DNA content induced by Geminin deficiency (Fig. 1C,D). These studies indicate that reduced expression of WDR5 is sufficient to suppress DNA re-replication in Geminin-deficient cells.

### The MLL-WDR5-RBBP5 methyltransferase complexes are required for re-replication

**Fig. 2.**
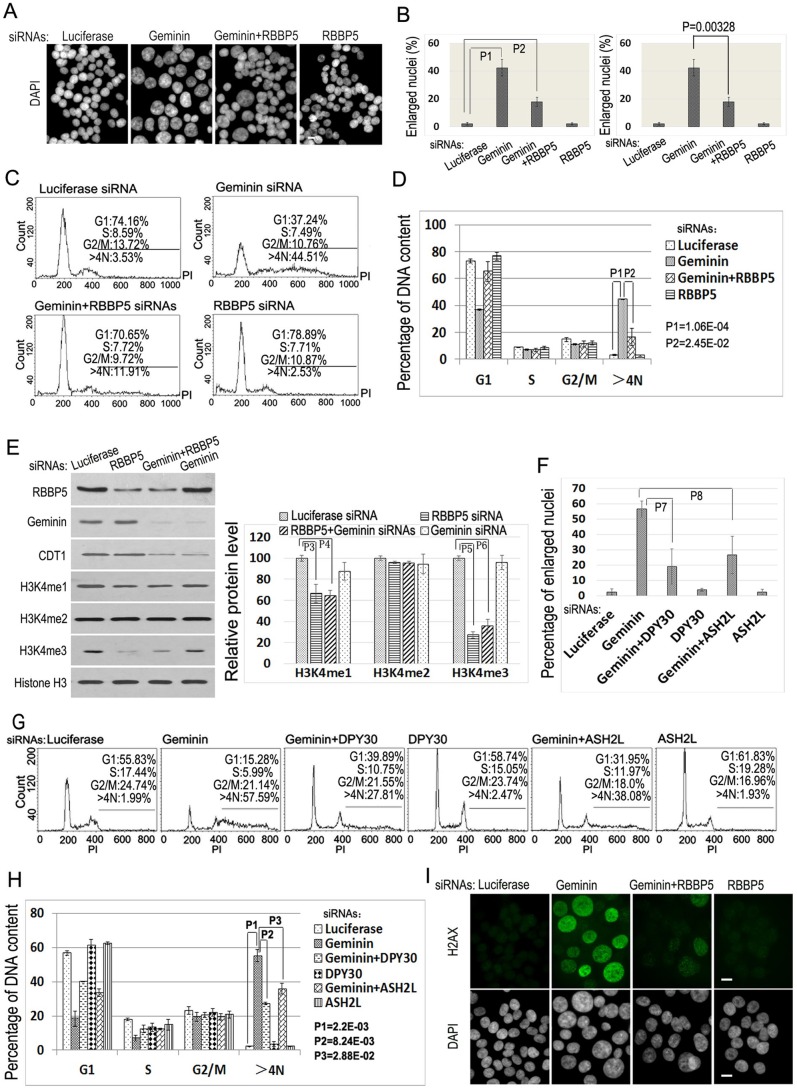
**DNA re-replication requires the components of the MLL complexes.** (A) HCT116 cells were transfected with 50 nM siRNAs of luciferase, Geminin, Geminin+RBBP5, and RBBP5 for 48 h. The nuclei were stained with DAPI. Scale bar: 50 μm. (B) The percentages of enlarged nuclei in various siRNA-ablated cells in A. Enlarged nuclei were examined, quantified, and error bars calculated in each sample for standard deviations as in Fig. 1B. Left plot: the statistical differences in enlarged nuclei between control siRNA-treated and each specific siRNA-treated cells were calculated using the two-tailed Student's *t*-test (*P*1=3.17E-04 and *P*2=1.39E-03). Right plot: significant difference was observed between Geminin siRNA-treated and Geminin+RBBP5 double siRNA-treated cells (P=0.00328<0.01) as indicated. (C) FACS analysis of DNA contents in A. The polyploid cell populations (>4N) are indicated. Three independent experimental repeats were conducted with similar results, and only one representative experiment is shown. (D) The statistical differences in polyploid DNA (>4N) in Geminin siRNA- treated and Geminin+RBBP5 double siRNA-treated cells were calculated using the two-tailed Student's *t*-test (*P*1=1.06E-04, *P*2=2.45E-02). (E) The proteins in the lysates from siRNA-treated cells in A were analyzed by immunoblotting with specific antibodies as indicated. The changes of histone H3K4 methylations were quantified on the right plot. Error bars indicate standard deviation of triplicated samples. The statistical differences were evaluated between control (luciferase) and specific siRNA samples using the two-tailed Student's *t*-test (*P*3=0.0335, *P*4=0.00397, *P*5=0.0233, *P*6=0.0168, *P*7=7.61E-04 and *P*8=0.0020). (F) Reduction of DPY30 or ASH2L suppresses the formation of polyploid nuclei in Geminin-deficient cells. HCT116 cells were transfected with 50 nM siRNAs of luciferase, Geminin, DPY30 or ASH2L, and Geminin+DPY30 or ASH2L for 48 h as in A. The statistical significance between single Geminin siRNA- treated and Geminin+DPY30/ASH2L double siRNA-treated cells was analyzed by the two-tailed Student's *t*-test (*P*9=0.00663 and *P*10=0.0171). (G) FACS analysis of DNA content from cells in F. The polyploid DNA fractions of cells were indicated. Three independent experimental repeats were conducted with similar results, and only one representative experiment is shown. (H) The statistical differences in polyploid DNA (>4N) in Geminin siRNA- treated and Geminin+DPY30 or ASH2L double siRNA-treated cells were calculated as in 2D. (I) Immunofluorescence staining of H2AX in A. Scale bars: 50 μm.

Because WDR5 acts as an essential component of the MLL methyltransferase complexes (Wysocka et al., 2005), we also monitored the effects of WDR5 downregulation on H3K4 methylations in parallel. The reduction of WDR5 expression significantly downregulated the levels of mono- and trimethylated H3K4, and was consistent with previous reports (H3K4me1 and H3K4me3, Fig. 1E,F) (Wysocka et al., 2005). To further test whether the MLL methyltransferase complexes are involved in re-replication, we examined the silencing effects of RBBP5 (Wysocka et al., 2005), another key component of the MLL complexes, on DNA re-replication. We found that co-silencing of RBBP5 and Geminin by specific siRNAs also markedly reduced the formation of enlarged nucleus in Geminin-deficient cells (Fig. 2A,B) and suppressed the percentage of cells containing >4N DNA induced by Geminin deficiency alone in (Fig. 2C,D). The siRNA-mediated reduction of RBBP5 also significantly decreased the number of cells that are positive for H2AX staining in Geminin-deficient cells (Fig. 2I), which is associated with reduced levels of both mono- and trimethylated H3K4 (Fig. 2E).

Because multiple MLL proteins exist to form various MLL complexes (Greer and Shi, 2012), it is difficult to simultaneously ablate multiple MLL family members in a single cell. Therefore, we tested the involvement of ASH2L and DPY30, other components of the MLL protein complexes (Greer and Shi, 2012; Jiang et al., 2011; Wysocka et al., 2005), to determine the roles of the MLL complexes in DNA re-replication. Our studies revealed that reduced expression of either ASH2L or DPY30 by specific siRNAs also led to the suppression of DNA re-replication in Geminin-deficient cells (Fig. 2G,H). These studies indicate that the MLL-RBBP5-WDR5 complexes and their catalytic activities towards H3K4 methylation are involved in DNA re-replication.

### Alpha-amanitin does not suppress DNA re-replication

**Fig. 3.**
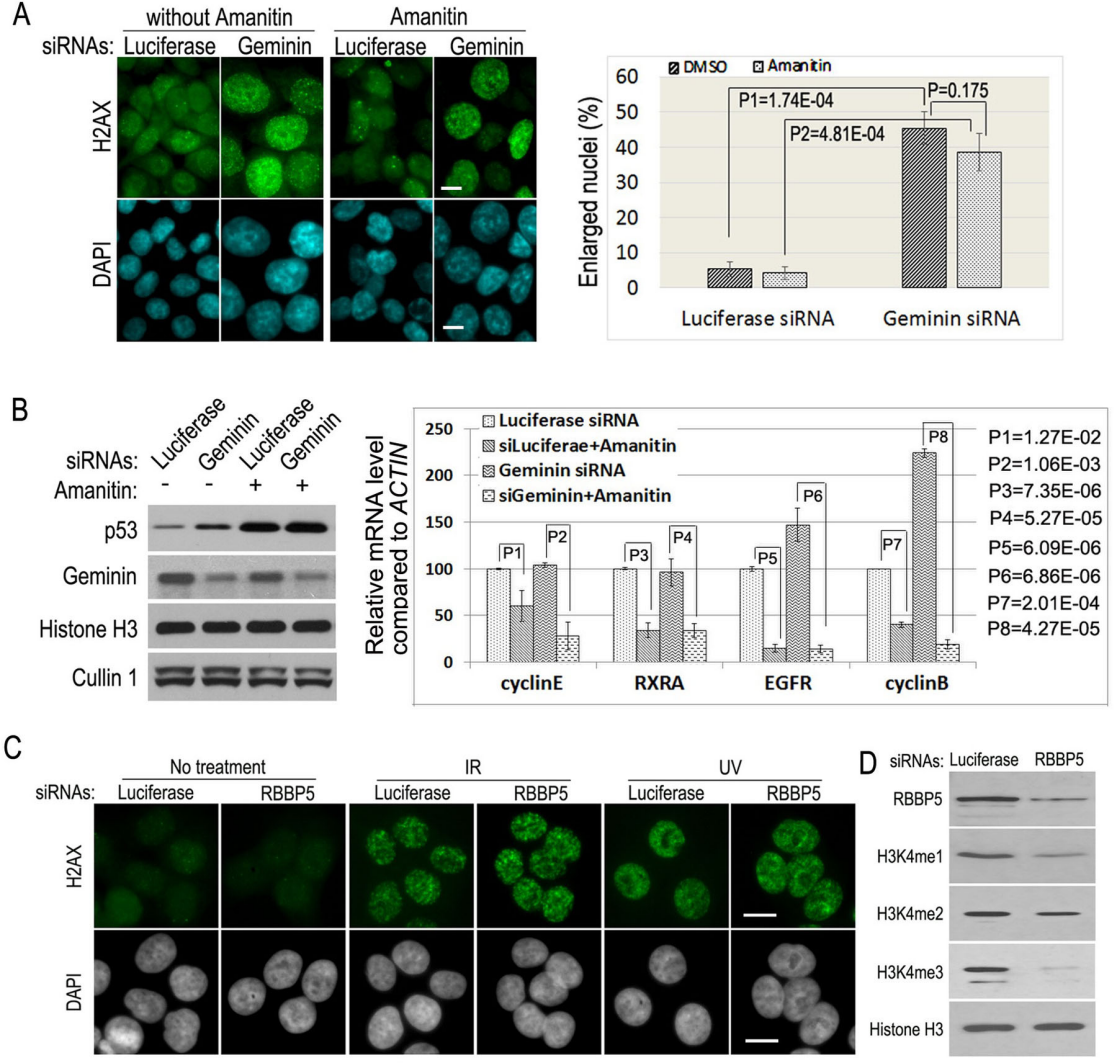
**Downregulation of the components of the MLL complexes does not affect DNA damage response.** (A) DNA re-replication induced by Geminin deficiency is not sensitive to transcriptional inhibitor α-amanitin. HCT116 cells were transfected with 50 nM siRNAs of luciferase and Geminin with or without α-amanitin (2.5 μg/ml). The cells were fixed 24 h later and assayed for H2AX staining (left panel) and the percentages of enlarged nuclei (right panel). Enlarged nuclei were calculated as described in Fig. 1B. Significant difference was observed between control (luciferase) siRNA-treated and Geminin siRNA-treated cells. *P*-value was assessed using the two-tailed Student's *t*-test (*P*5=1.74E-04 and *P*6=4.81E-04). There is no statistically significant difference on the formation of enlarged nuclei between α-amanitin-treated and control (DMSO)-treated cells, which was evaluated by the two-tailed Student's *t*-test (*P*=0.175>0.05). (B) Analysis of α-amanitin effects on the induction of p53 by western blotting (left panels) and on the mRNA levels of cyclin E, RXRA, EGFR, and cyclin B by quantitative real-time reverse transcription PCR (right panel) in A. The statistical differences in control and α-amanitin-treated cells were calculated using the two-tailed Student's *t*-test. (C,D) Reduction of RBBP5 has no effects on H2AX staining induced by X-ray (IR) or UV irradiation. Scale bars: 50 μm. Cells were transfected with 50 nM luciferase and RBBP5 siRNAs and, after 48 h, were treated with 10 Gy X-ray or 10 J/cm^2^ UV. The cells were fixed 30 min later for H2AX staining (C) and western blotting (D).

The MLL-WDR5-RBBP5 methyltransferase complexes regulate the levels of methylated H3K4, which are usually associated with transcriptionally active regions on chromatin (Greer and Shi, 2012). To test whether inhibition of transcription blocks re-replication, we examined whether α-amanitin (Chafin et al., 1995), an inhibitor of RNA polymerase II-mediated transcription, produces the same suppressive effects as that of WDR5 or RBBP5 deficiency on DNA re-replication. We found α-amanitin did not significantly suppress the formation of polyploid and enlarged nuclei in Geminin siRNA-depleted cells, even though α-amanitin indeed reduced the mRNA levels of cyclin E, retinoic acid receptor-alpha (RXRA), EGFR, and cyclin B, as well as induction of an elevated level of the p53 protein, as previously reported for the transcription inhibitory effects of this compound (Fig. 3A,B) (Ljungman et al., 1999). Our studies indicate that the MLL-WDR5-RBBP5 methyltransferase complexes are directly involved in regulating this specific type of DNA replication.

### RBBP5 is not required for DNA damage response

Since DNA re-replication induces the fork structures during DNA replication elongation that activates the DNA damage checkpoint response and consequently promotes the formation of H2AX nuclear foci (Jin et al., 2006; Melixetian et al., 2004), we wondered whether the MLL complexes are also required for the DNA damage checkpoint activation. Our studies indicate that although ultraviolet (UV) or X-ray irradiation induced intensive nuclear staining of H2AX (Fig. 3C), the siRNA-mediated reduction of RBBP5 and its-associated suppression of the mono- and trimethylations of H3K4 did not affect the H2AX nuclear staining in UV or X-ray irradiated cells (Fig. 3C,D). These analyses indicate that the siRNA-mediated silencing of RBBP5 expression and reduced activities of the MLL complexes on DNA re-replication are not due to their potential effects on the DNA damage checkpoint control.

### Reduction of RBBP5 suppresses DNA re-replication in CDT2-deficient cells

**Fig. 4.**
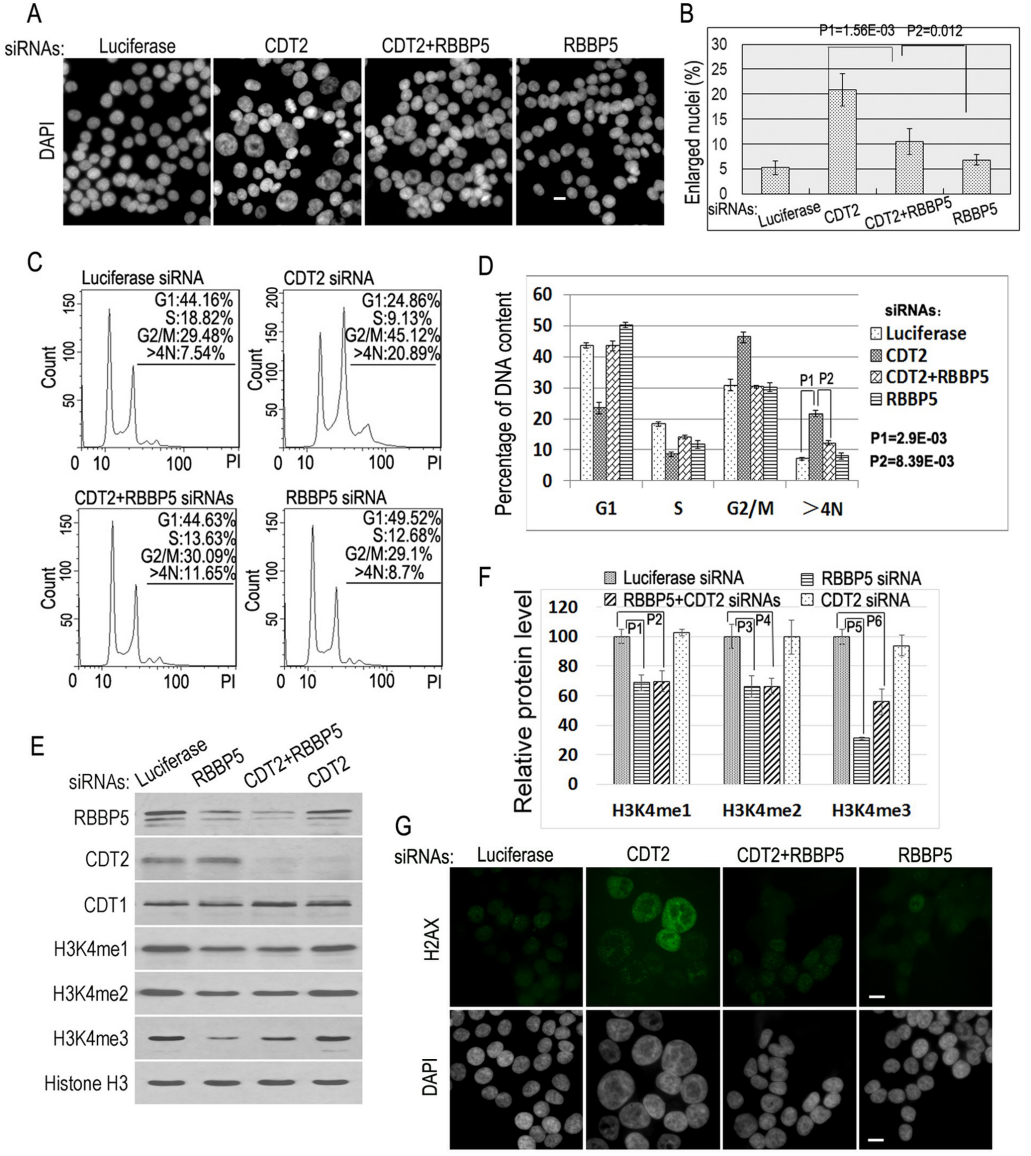
**Inactivation of RBBP5 blocks re-replication induced by CDT2 deficiency.** (A) HCT116 cells were transfected with 50 nM siRNAs of luciferase, CDT2, CDT2+RBBP5, and RBBP5. The nuclei were stained with DAPI. Scale bar: 50 μm. (B) The percentages of enlarged nuclei in various siRNA-ablated cells in A. Enlarged nuclei were examined, quantified, and error bars calculated in each sample for standard deviations as in Fig. 1B. The statistical differences between control and specific siRNA samples were calculated using the two-tailed Student's *t*-test (*P*1=1.56E-03<0.05). The statistical difference was analyzed between CDT2 siRNA-treated and CDT2+RBBP5 double siRNA-treated cells using the two-tailed Student's *t*-test (*P*2=0.012<0.05) as indicated. (C) FACS analysis of DNA contents in A. Three independent experimental repeats were conducted with similar results, and only one representative experiment is shown. (D) The statistical differences in polyploid DNA (>4N) in CDT2 siRNA-treated and CDT2+RBBP5 double siRNA-treated cells were calculated using the two-tailed Student's *t*-test (*P*1=2.9E-03, *P*2=8.39E-03). (E) The proteins from the lysates of cells in A were analyzed by immunoblotting with specific antibodies as indicated. (F) Relative protein levels of histone H3K4 mono-, di- and trimethylation (H3K4me1-3) in D were quantified as in Fig. 1F. The error bars indicate standard deviation of triplicated samples. Statistical significances were performed with the two-tailed Student's *t*-test by comparing between control (luciferase) and specific siRNA-treated samples (*P*1=0.00252, *P*2=0.00948, *P*3=0.0127, *P*4=0.0073, *P*5=8.19E-05 and *P*6=0.00508). (G) Immunofluorescence staining of H2AX in A. Scale bars: 50 μm.

To further rule out the possibility that MLL complexes affect a Geminin-dependent but replication-independent process in our studies (Kroll et al., 1998), we also examined the requirement of MLL complexes in DNA re-replication induced by the decreased levels of the CLR4^CDT2^ ubiquitin E3 ligase complex. Reduced expression of CDT2 by specific siRNAs stabilizes the CDT1 protein and consequently induces chromosomal re-replication and polyploidy (Fig. 4A-D) (Havens and Walter, 2011; Higa et al., 2006a,b; Jin et al., 2006). Our examination revealed that co-silencing of RBBP5 and CDT2 led to the suppression of CDT2 deficiency-induced DNA re-replication, including reduced formation of enlarged nuclei, decreased cell population containing >4N DNA content by FACS analyses, and inhibition of the H2AX staining (Fig. 4). This RBBP5 deficiency-induced suppression of re-replication is associated with the concomitant reduction of mono- and trimethylated H3K4 (Fig. 4E,F). Thus, our studies indicate that the MLL complexes are required for DNA re-replication, a specific type of DNA replication, induced by silencing of either Geminin or CDT2 expression.

### Downregulation of RBBP5 or WDR5 blocks the loading of MCM2-7 onto chromatin

**Fig. 5.**
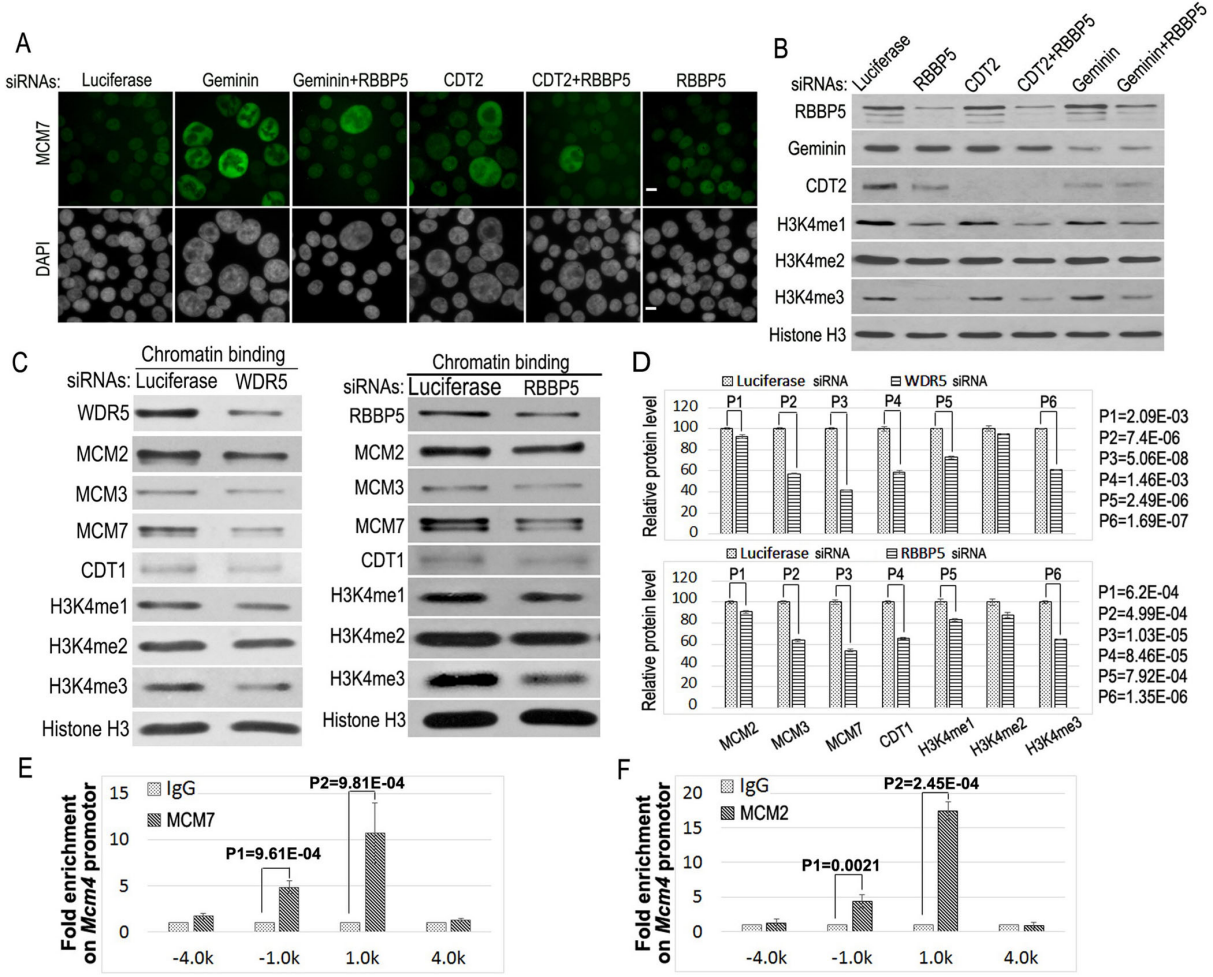
**Inactivation of the MLL complex prevents the recruitment of MCM2-7 proteins onto chromatin.** (A) HCT116 cells were transfected with 50 nM siRNAs of luciferase, Geminin, Geminin+RBBP5, CDT2, CDT2+RBBP5 and RBBP5. The cells were fixed and stained with anti-MCM7 and FITC-conjugated secondary antibodies and counter-stained with DAPI. Scale bars: 50 μm. (B) Proteins from the siRNA-treated cell lysates in A were analyzed by specific antibodies as indicated. (C) Downregulation of WDR5 or RBBP5 reduces the recruitment of MCM proteins to chromatin. Chromatin fractions were isolated from control and WDR5 or RBBP5 siRNA-treated cells and the chromatin-associated MCM proteins were examined by western blotting as indicated. (D) Relative protein levels of MCMs and methylated histone H3K4 on chromatin in C were quantified using Gel-Pro analyzer 4.0. The error bars indicate standard deviation of triplicated samples. The statistical differences of MCM2, MCM3, MCM7, CDT1 and histone H3K4 methylations between control and specific siRNA samples were analyzed using the two-tailed Student's *t*-test. (E,F) The chromatin immunoprecipitation (ChIP) analysis was performed to locate MCM7 (E) and MCM2 (F) proteins on the DNA replication origin at the *Mcm4* gene. Proteins were cross-linked to chromatin and chromatin DNA was sonicated to generate 500-1000 base-pairs (bps) fragments in average length. The ChIP-grade anti-MCM7 and MCM2 antibodies were used for chromatin immunoprecipitation. Cross-linked DNA was released, purified, and analyzed for the enrichment of DNA fragments associated with MCM2 and MCM7 from −4.0 kb to 4.0 kb (kilobase pairs) along the *Mcm4* region using various Mcm4 primers and quantitative real time PCR as described in the Materials and Methods. Error bars indicate the standard deviation of triplicated samples. The statistical significance of antibody-enriched specific *Mcm4* DNA sequences over the background control IgG binding (fold enrichment) was assessed using the two-tailed Student's *t*-test.

The key event of DNA replication licensing by the activated CDT1 is the loading of the MCM2-7 complex, a replicative DNA helicase complex, onto chromatin to assemble the pre-RC for DNA replication initiation at the replication origins (Arias and Walter, 2007; Ballabeni et al., 2004; Higa et al., 2003; Wong et al., 2010). To determine whether downregulation of the MLL complexes affects this critical replication event, we examined the recruitment of MCM7 into the nucleus, a replication licensing dependent event (Fig. 5A) (Higa et al., 2003). While low levels of nuclear MCM7 staining in control cells were weakly detectable, silencing of Geminin or CDT2 promoted strong nuclear staining of MCM7 in enlarged re-replicating nuclei (Fig. 5A) (Higa et al., 2003). However, co-silencing of RBBP5 eliminated most of the intensive nuclear staining of MCM7 and the formation of enlarged nuclei in Geminin or CDT2-deficient cells. This suppression is associated with the concomitant downregulation of mono- and trimethylated H3K4 (Fig. 5A,B). We also examined whether reduced expression of WDR5 or RBBP5 in the MLL complexes affects the MCM2-7 recruitment to chromatin using biochemical fractionation. Our studies revealed that siRNA-mediated silencing of either WDR5 or RBBP5 expression reduced the association of MCM2, MCM3, MCM7 and CDT1 with the fractionated chromatin (Fig. 5C,D), indicating that the MLL-WDR5-RBBP5 complexes are required for the recruitment of MCM2-7 and CDT1 to chromatin for DNA replication.

### The MLL complexes and methylated H3K4 co-exist at the *Mcm4* replication origin

**Fig. 6.**
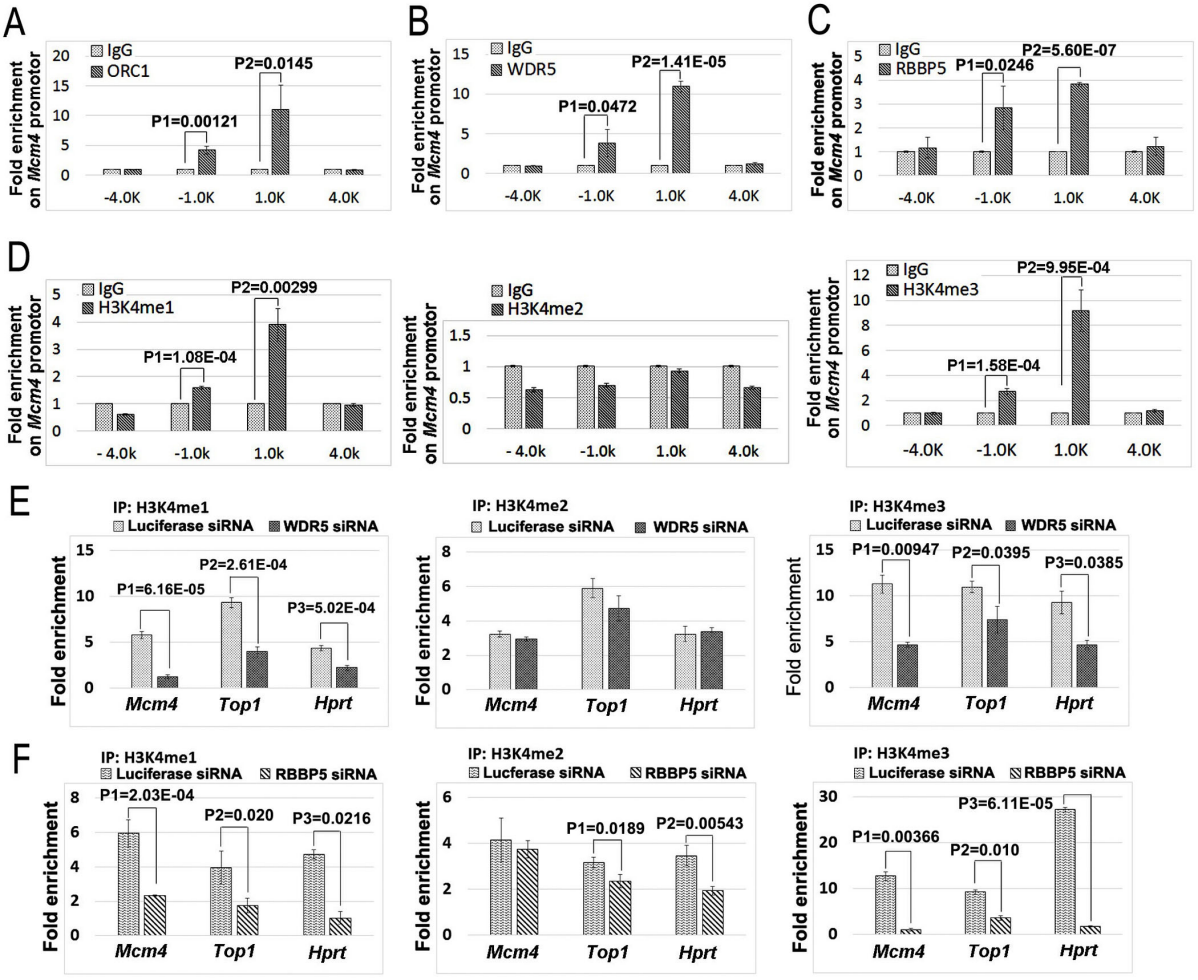
**WDR5, RBBP5 and methylated H3K4 co-localize with ORC1 at replication origins.** (A-C) WDR5 and RBBP5 are enriched in the replication origins at the *Mcm4* gene. The ChIP analysis was performed to locate ORC1 (A), WDR5 (B), and RBBP5 (C) on the DNA replication origin at the *Mcm4* gene as in Fig. 5E,F. Experiments were performed in triplicates and the error bars indicate the standard deviation of triplicated samples. The statistical significance of antibody-enriched specific *Mcm4* DNA sequences over the background control IgG binding (fold enrichment) was assessed using the two-tailed Student's *t*-test. (D) Methylated forms of histone H3K4 were enriched on the *Mcm4* replication origin region. The location of histone H3K4me1/2/3 on the *Mcm4* gene was mapped by ChIP using anti-H3K4me1/2/3 antibodies, respectively. Error bars indicate standard deviation of triplicated samples. The statistical differences in the enrichment of specific DNA sequences over the background IgG binding were analyzed as in A-C using the two-tailed Student's *t*-test. (E,F) Reduction of WDR5 (E) or RBBP5 (F) down-regulates methylated H3K4 on DNA replication origins at the *Mcm4*, *Top1* and *HPRT* genes, as analyzed by ChIP. The statistical significances of the differences in various methylated forms of H3K4 between control luciferase siRNA-treated and WDR 5/RBBP 5 siRNA-treated HCT116 cells were examined using the two-tailed Student's *t*-test. Error bars indicate standard deviation of triplicated samples.

It is well established that DNA replication licensing occurs at defined replication origins that are marked by the presence of the ORC1-6 and MCM2-7 complexes. The firing of early DNA replication origins often associates with actively transcribed regions, which are usually associated with methylated H3K4 (Arias and Walter, 2007; Karnani et al., 2010). We sought to determine whether the MLL complexes and methylated H3K4 are associated with DNA replication origins, such as the well-characterized origin region at the human *Mcm4* gene (Miotto and Struhl, 2008, 2010; Schaarschmidt et al., 2002). We used specific antibodies against MCM2, MCM7, and ORC1 to help locate the chromosomal origin regions associated with the *Mcm4* gene using the chromatin-immunoprecipitation analysis (ChIP) (Miotto and Struhl, 2008; Schaarschmidt et al., 2002). Consistent with previous reports (Miotto and Struhl, 2008, 2010; Schaarschmidt et al., 2002), we repeatedly found that both MCM2 and MCM7 proteins were enriched in the −1.0 and +1.0 kb regions of the *Mcm4* gene, with a prominent peak near the +1.0 kb region, relative to the transcription start region (Fig. 5E,F). Using the MCM2 and MCM7 binding regions as the reference, we also analyzed the distribution of ORC1, WDR5 and RBBP5 along the *Mcm4* gene. Our ChIP analyses revealed that ORC1, WDR5, and RBBP5 are also specifically enriched within the same −1.0 and +1.0 kb regions of the *Mcm4* gene, in particular near the +1.0 kb region (Fig. 6A-C), overlapping that of MCM2 and MCM7. In addition, the mono- and trimethylated H3K4 forms, but not dimethylated H3K4, are enriched at the −1.0 and +1.0 kb regions of the *Mcm4* gene (Fig. 6D) co-localizing again with that of ORC1, MCM2-7, WDR5 and RBBP5 binding regions, suggesting that MLL complexes and H3K4 methylation are likely involved in the control of DNA replication origins.

### The MLL-regulated histone H3K4 methylation is required for the association of MCM2-7 complexes with DNA replication origins on chromatin

**Fig. 7.**
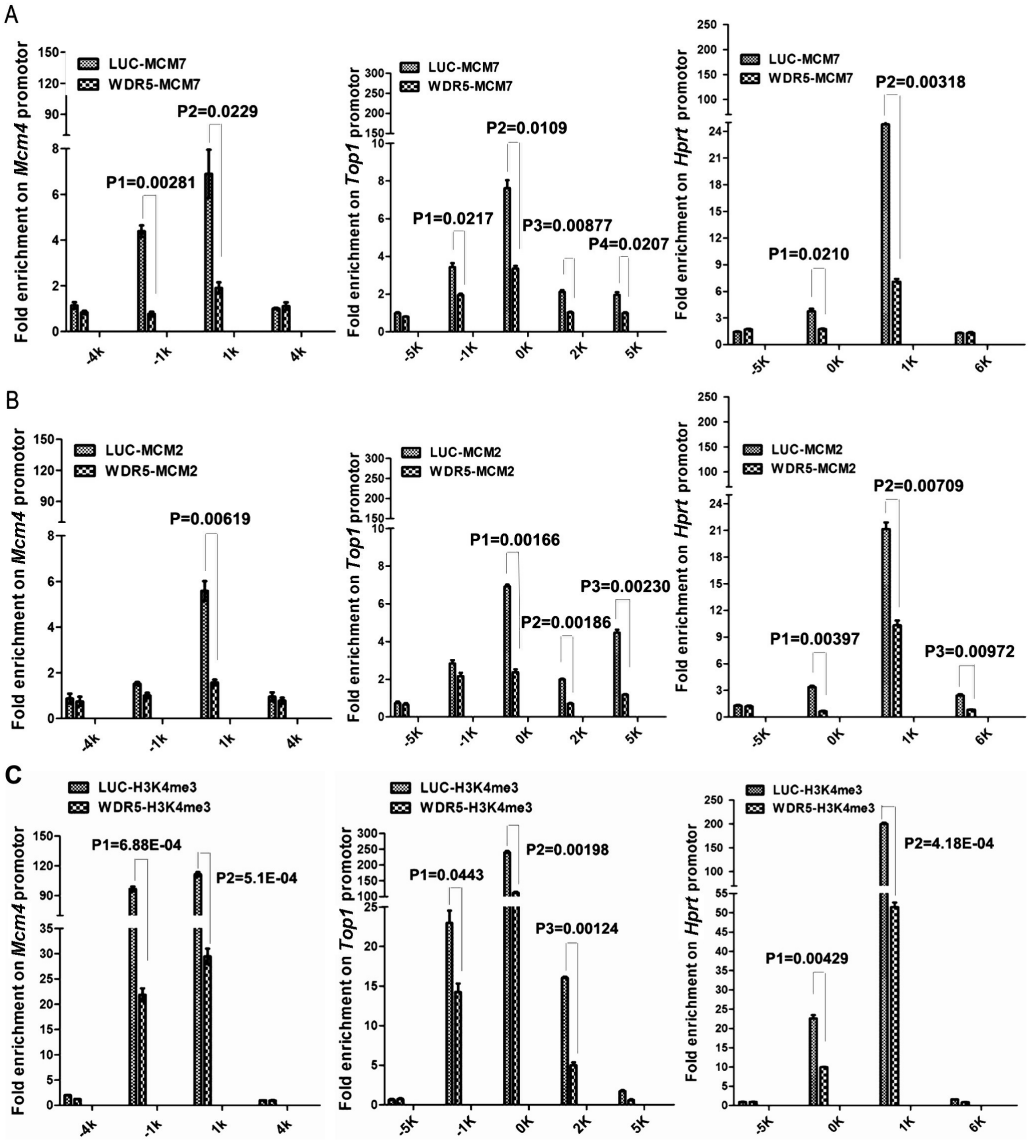
**Downregulation of WDR5 reduces the recruitment of MCM2 and MCM7 onto replication origins.** HCT116 cells were transfected with 50 nM siRNAs of luciferase or WDR5 for 48 h and the association of MCM7 (A), MCM2 (B), and trimethylated H3K4 (C) at the *Mcm4*, *Top1* and *HPRT* genes were analyzed by ChIP. Error bars indicate standard deviation of triplicated samples. The statistical significances of the differences between control and WDR 5-ablated cells were analyzed using the two-tailed Student's *t*-test. Error bars indicate standard deviation of triplicated samples.

We also determined whether the methylated H3K4 is associated with other established replication origins in human cells (Cohen et al., 2002; Keller et al., 2002; Miotto and Struhl, 2008, 2010; Schaarschmidt et al., 2002). Our ChIP analyses confirmed that the methylated H3K4 is also associated with replication origins associated with the *Top1* and *HPRT* genes (Fig. 6E,F), which are co-localized with both MCM2 and MCM7 proteins (Fig. 7A-C). To determine whether the origin-associated H3K4 methylations are dynamically regulated by the MLL-WDR5-RBBP5 methyltransferase complexes, we reduced the expression of WDR5 or RBBP5 by specific siRNAs and monitored the responses on various methylated forms of H3K4 in these replication origins. We found that reduction of WDR5 or RBBP5 significantly downregulates the levels of the mono- and trimethylated H3K4 at the origin regions at *Mcm4*, *Top1*, and *HPRT* (Fig. 6E,F), consistent with the specificity of the MLL-WDR5-RBBP5 methyltransferase complexes that catalyze these methylated forms of H3K4.

Because the MCM2-7 complexes are also co-localized with the methylated H3K4 on Mcm4, *Top1* and *HPRT* genes (Figs 5E,F and 7A-C), we determined whether the association of the MCM2-7 complex to these replication origins is affected by downregulation of the MLL complexes such as WDR5 and consequent reduction of methylated H3K4. Notably, our studies revealed that siRNA-mediated silencing of WDR5 expression led to the reduced recruitment of MCM2 and MCM7 proteins onto the origin regions at the *Mcm4, Top1*, and *HPRT* genes (Fig. 7A,B), which coincided with the downregulation of H3K4me3 (Fig. 7C). Thus, our studies indicate that the MLL complexes and the methylated H3K4 are required for the association of MCM2 and MCM7 to these replication origins.

## DISCUSSION

In this report, we found that the siRNA-mediated reduction of RBBP5, WDR5, ASH2L, or DPY30 (components of the MLL methyltransferase complexes), all inhibited a specific type of DNA replication: the chromosomal DNA re-replication induced by downregulation of Geminin or CDT2 in HCT116 cells (Figs 1, 2 and 4). Although re-replication induces the formation of DNA replication elongation fork structures that activate DNA replication/DNA damage checkpoints, as revealed by H2AX staining in Geminin or CDT2-deficient cells, the MLL complexes are not directly required for UV- or X-ray-induced DNA damage checkpoints (Fig. 3). Since the MLL histone methyltransferase complexes catalyze the mono- and trimethylation on H3K4 (Greer and Shi, 2012; Jiang et al., 2011; Wysocka et al., 2005), our studies provide strong evidence that histone methylations on H3K4 by the MLL complexes are required for this specific type of DNA replication.

Many early studies have shown that DNA replication in early S-phase is associated with transcriptionally active regions that are typically marked by methylated H3K4. However, it is not clear whether methylated H3K4 is directly involved in the control of origin-dependent replication. Our additional studies provide strong evidence that the MLL complexes and methylated H3K4 are directly involved in DNA replication. In support of this notion, our studies have shown that both the components of the MLL complexes and the methylated H3K4 are associated with several well-characterized DNA replication origins at the *Mcm4*, *Top1*, and *HPRT* genes, which are marked by the presence of the ORC and MCM2-7 complexes (Figs 5-7). Notably, depletion of the components of MLL complexes such as WDR5 or RBBP5, which caused the downregulation of the mono- and trimethylated H3K4 at replication origins (Figs 6 and 7), diminished the recruitment of the MCM2-7 complex to chromatin (Fig. 5A,B and Fig. 7A-C). It is well established that the methylated H3K4 may provide an open chromatin conformation for transcription. It is likely that the MLL-regulated methylation of H3K4 and the consequent open chromatin conformation may also be required for DNA re-replication. Our work provides strong evidence that the MLL complexes and methylated H3K4 are directly involved in DNA replication through the replication licensing process.

So far, it has been shown that silencing of the expression of acetylase HBO1 can suppress DNA re-replication (Miotto and Struhl, 2008, 2010). Our studies suggest that the involvement of H3K4 methylation in DNA replication is likely to be independent of the HBO1-mediated pathway. Although HBO1 forms a complex with JADE1/2/3 and ING4/5 (Saksouk et al., 2009) and co-exists with the ORC complex at DNA replication origins, ING4/5 is not found in association with DNA replication origins (Miotto and Struhl, 2008). HBO1 is shown to bind chromatin through its interaction with JADE1/2/3, which interacts with un-modified N-terminus of H3K4 through the PHD domains of JADE1/2/3. However, the binding of HBO1-JADE1/2/3 to the N-terminus of histones is much reduced if H3K4 is methylated (Saksouk et al., 2009). Thus, our finding that the MLL-RBBP5-WDR5 mediated-methylation of H3K4 is essential for DNA replication represents an independent histone modification required for DNA replication licensing on chromatin.

## MATERIALS AND METHODS

### Antibodies, cells and transfection

Anti-p53 (Sc-126), Mcm7 (Sc-9966) and actin (Sc-1616) antibodies were purchased from Santa Cruz Biotechnologies (Dallas, TX). Anti-histone H3 (ab1791), H3K4me1 (ab8895), H3K4me2 (ab32356), H3K4me3 (ab8580), ORC1 (ab60), ASH2L (ab50699), MCM2 (ab4461), and DPY30 (ab126352) antibodies were from Abcam (San Francisco, CA). The anti-phospho-histone H2AX (Ser139) antibody (#2577) was purchased from Cell Signaling (Danvers, MA). Anti-Geminin (A300-935A), CDT1 (A300-786A), CDT2 (A300-948A), MCM2 (A300-191A), WDR5 (A302-430A), and RBBP5 (A300-109A) antibodies were purchased from Bethyl Laboratories Inc. (Montgomery, TX). Fluorescein isothiocyanate (FITC)-conjugated rabbit secondary antibodies (111-097-003) were from Jackson ImmunoResearch Laboratories (West Grove, PA). Anti-CUL1 antibody was described previously (Yin et al., 2014; Zhang et al., 2013). For siRNA-mediated silencing, human colorectal carcinoma HCT116 cells were transfected with 50 nM siRNAs for 48 h as previously described (Higa et al., 2006b; Zhang et al., 2013). The sequences of the siRNAs are: Geminin: AAUGCCAACUCUGGAAUCA; RBBP5: GAGCCGAGAUGGUCAUAAAUU; CDT2: ACTCCTACGTTCTCTATTA; WDR5: CAGAGGATAACCTTGTTTA; DPY30: CAGCUUUAAUUGCCAUGAU; ASH2L: CAAGGACUUUCUGGGAAAUA; and Luciferase (Luc): CGTACGCGGAATACTTCGA. Immunostaining was conducted using specific antibodies as previously described (Higa et al., 2003).

### Cell culture and flow cytometry

HCT116 cells were purchased from ATCC (CCL-247) and cultured in McCoy's 5a Medium supplemented with 10% fetal bovine serum and 1% antibiotics (Invitrogen). The cells have been recently authenticated and tested for contamination based on the pseudo-diploid genome and protein markers. For flow cytometry (FACS) analysis, cells were harvested by trypsinization and fixed in 70% ethanol at 4°C for 2 h. They were washed again in 1XPBS and incubated with 25 μg/ml propidium iodide (PI) staining buffer containing 1% TrionX-100 and 50 μg/ml RNAase for 30 min at 37°C and analyzed by FACS (Cytomis FC 500, Beckman Coulter), and evaluated with the CXP software as described previously (Mihaylov et al., 2002; Zhang et al., 2013). Cell Growth analysis by MTT was conducted as described previously (Zhang et al., 2013).

### Immunostaining and chromatin association

Cells were cultured on cover slips in 35 mm dishes and were fixed with 3.7% paraformaldehyde for 15 min and then permeabilized with 0.3% Triton X-100 for 10 min on ice (Zhang et al., 2013). Cells were incubated with the primary antibodies overnight at 4°C, washed, and stained for an hour with fluorescence-labeled secondary antibodies at room temperature, as described previously (Higa et al., 2003). Cover slips were mounted with Mowoil containing 1 μg/ml 4′, 6-diamidino-2-phenylindole (DAPI) for DNA staining. Images were captured on an Olympus fluorescence microscope (Olympus CKX41, Japan) coupled to a cooled charge-coupled device camera (QICAM, Japan) and processed by using the QCapture Pro 6.0 program (Zhang et al., 2013). For transcriptional inhibitory assays, α-amanitin (2.5 μg/ml) was added to the medium together with siRNAs and the cells were fixed 24 h after transfection. Phosphorylated H2AX was detected by anti-H2AX-Ser139 phosphorylation antibody. For analysis of the recruitment of MCM2-7 and other proteins, chromatin was isolated by fractionation and chromatin-associated proteins were detected by western blotting as described by Mendez and Stillman (2000).

### Chromatin immunoprecipitation (ChIP)

Chromatin immunoprecipitation (ChIP) was conducted according to a protocol described previously (Miotto and Struhl, 2008; Zhang et al., 2013). Briefly, 2×10^7^ cells were used for each sample. Proteins were cross-linked to DNA by adding formaldehyde to a final concentration of 0.75% and the cross-linking was terminated by 125 mM glycine. Cells were harvested and resuspended in the FA lysis buffer (50 mM HEPES-KOH pH 7.5, 140 mM NaCl, 1 mM EDTA pH 8.0, 1% Triton X-100, 0.1% sodium deoxycholate, 0.1% SDS and protease inhibitors) and sonicated to generate DNA fragments. Soluble chromatin was diluted eight times with RIPA buffer (50 mM Tris-HCl pH 8.0, 150 mM NaCl, 2 mM EDTA pH 8.0, 1% NP-40, 0.5% sodium deoxycholate, 0.1% SDS and protease inhibitors) and pre-cleared with protein A sepharose beads. Primary antibodies were then added and incubated with the chromatin fragments overnight at 4°C and captured by protein A sepharose beads for 2 h. The immunocomplexes were washed three times with the washing buffer (0.1% SDS, 1% Triton X-100, 2 mM EDTA pH 8.0, 150 mM NaCl and 20 mM Tris-HCl pH 8.0) and once with final washing buffer (0.1% SDS, 1% Triton X-100, 2 mM EDTA pH 8, 500 mM NaCl and 20 mM Tris-HCl pH 8.0). Immunocomplexes were eluted from protein A beads in elution buffer (1% SDS and 0.1 M NaHCO3) and cross-linking was reversed at 65°C for 5 h. DNA was extracted with phenol/chloroform and finally precipitated with 100% ethanol. Purified DNA was quantified by real-time PCR using SYBR green on an ABI Prism 7300 System. Primers used are listed in Table S1.

### Quantification and statistical analyses

The nuclei with diameters more than 70 μm in Geminin and CDT2 siRNA-ablated and other related cells were considered as re-replicated enlarged nuclei as compared to the nuclei in control cells (luciferase siRNA), which have an average nucleus diameter about 40-50 μm (Ballabeni et al., 2004; Jin et al., 2006; Mihaylov et al., 2002). To obtain statistically significant percentages of enlarged nuclei in each sample, nuclei in five independent microscopic fields were scored, with 300 nuclei counted in each field (total 1500 nuclei for each sample). The ratios of enlarged to normal nuclei were averaged from five counts and plotted with error bars representing standard deviation (Mihaylov et al., 2002). The statistical differences between control and each specific siRNA-treated cells were calculated using the two-tailed Student's *t*-test to generate the *P*-value (Fay and Gerow, 2013). Similarly, the statistical differences between samples such as the comparison between single siRNA- and double siRNA-silenced (co-silenced) cells were also measured by the two- tailed Student's *t*-test, and *P*<0.05 was considered statistically significant (Fay and Gerow, 2013). The results of each silencing experiment were confirmed by at least three independent repeats.

To compare the relative protein levels on western blots, the gel analysis software Gel-pro analyzer 4.0 (Media Cybernetics) was used to extract qualitative and quantitative information on protein bands from each western blot (Fay and Gerow, 2013). The mean density of each protein band from the software-derived output was first normalized with that of protein loading control in the same sample. The relative protein level of each protein band was subsequently calculated by comparing its mean density with that of the control (luciferase siRNA) sample, which was set as 100% (Yin et al., 2014). Triplicated sample loadings were used to calculate mean±s.d., indicated as error bars. Statistical tests for significance were done with the two- tailed Student's *t*-test compared to control and *P*<0.05 was considered statistically significant (Fay and Gerow, 2013). All results are confirmed by at least three independent experiments.

For the ChIP assays, quantitative real-time PCR was used to quantify the enrichment of proteins and histone H3K4 methylation on specific DNA sequences of replication origins using the comparative Ct method as previously described (Miotto and Struhl, 2008, 2010). The Ct value of each sample was normalized to that of internal control GAPDH and the relative binding/occupancy of a specific protein or histone H3K4 methylation to a specific DNA sequence was further normalized to the background binding of control IgG as fold over IgG (Zhang et al., 2013). Experiments were performed in triplicates. The statistical significances of the enrichment were calculated using the two- tailed Student's *t*-test and *P*<0.05 was considered statistically significant (Fay and Gerow, 2013). All results are confirmed by at least three independent experiments.
